# Dual Crosslinked Ion-Based Bacterial Cellulose Composite Hydrogel Containing Polyhexamethylene Biguanide

**DOI:** 10.3390/membranes12090825

**Published:** 2022-08-24

**Authors:** Baramee Chanabodeechalermrung, Tanpong Chaiwarit, Sarana Rose Sommano, Pornchai Rachtanapun, Nutthapong Kantrong, Chuda Chittasupho, Pensak Jantrawut

**Affiliations:** 1 Department of Pharmaceutical Sciences, Faculty of Pharmacy, Chiang Mai University, Chiang Mai 50200, Thailand; 2 Plant Bioactive Compound Laboratory (BAC), Department of Plant and Soil Sciences, Faculty of Agriculture, Chiang Mai University, Chiang Mai 50200, Thailand; 3 Cluster of Research and Development of Pharmaceutical and Natural Products Innovation for Human or Animal, Chiang Mai University, Chiang Mai 50200, Thailand; 4 Cluster of Agro Bio-Circular-Green Industry (Agro BCG), Chiang Mai University, Chiang Mai 50100, Thailand; 5 Division of Packaging Technology, Faculty of Agro-Industry, Chiang Mai University, Chiang Mai 50100, Thailand; 6 Department of Restorative Dentistry, Faculty of Dentistry, Khon Kaen University, Khon Kaen 40002, Thailand; 7 Research Group of Chronic Inflammatory Oral Diseases and Systemic Diseases Associated with Oral Health, Faculty of Dentistry, Khon Kaen University, Khon Kaen 40002, Thailand

**Keywords:** bacterial cellulose, crosslinking, hydrogel, alginate, pectin

## Abstract

Composite bacterial cellulose (BC) based hydrogel with alginate (A) or pectin (P) or alginate and pectin was fabricated via a physical crosslinking technique using calcium chloride (CaCl_2_) solution and incorporated with polyhexamethylene biguanide (PHMB) as an effective antimicrobial drug by immersion method. After that, the physicochemical properties of all hydrogel formulations were characterized. The result showed that the formulations with PHMB performed better physicochemical properties than the hydrogel without PHMB. Fourier transform infrared spectroscopy (FT-IR) showed the interaction between PHMB and the carboxylic group of alginate and pectin. BC/A-PHMB hydrogel performed suitable mechanical strength, fluid uptake ability, water retention property, drug content, high integrity value, and maximum swelling degree. Moreover, in vitro cell viability of BC/A-PHMB hydrogel revealed high biocompatibility with human keratinocyte cell line (HaCaT) and demonstrated prolong released of PHMB in Tris-HCl buffer pH 7.4, while rapid release in phosphate buffer saline pH 7.4. BC/A-PHMB hydrogel demonstrated good anti-bacterial activity against *S. aureus* and *P. aeruginosa*. In conclusion, BC/A-PHMB hydrogel could be a potential dual crosslinked ion-based hydrogel for wound dressing with anti-bacterial activity.

## 1. Introduction

Cellulose presented in the plant cell wall as a major component is a natural biopolymer [1] composed of glucose molecules via 1,4-β-glycosidic bonds [2], and produced by several species, such as plants, algae, fungi, and bacteria [3]. Bacterial cellulose (BC) consists of wide ribbon-shaped fibrils with a diameter of less than 100 nm, which form a three-dimension (3D) network, and provides exclusive properties such as high-water holding capacity, crystallinity, and high purity [4,5,6]. BC has the same molecular and chemical structure as plant cellulose, but it lacks hemicellulose, and lignin, including biogenic products [5]. The most dominant and potential BC producer is *Komagataeibacter xylinus*, known as *Acetobacter xylinum* for many years [4,6] and other BC producer species are *Agrobacterium*, *Pseudomonas* and *Rhizobium* [5]. BC plays an important role as a biomaterial in drug delivery systems, scaffolds, tissue and organ regeneration and wound healing [6].

Wound dressing made of BC performed better properties than traditional wound dressings in maintaining a moist environment around the wound, reducing pain, accelerating tissue reepithelization, along with reducing scar formation [5,6]. Wound dressings that focus on keeping the wound from dehydration and support the wound healing process are available in films, hydrocolloids, and hydrogel [7]. Hydrogels are 3D network structures composed of crosslinked hydrophilic polymer that absorb large amounts of biological fluids or water in their swollen state [8,9]. The benefit of hydrogels over traditional wound dressing is the ability to increase the wound healing process depending on the wound stage [10]. Hydrogels provide properties that meet most of the criteria for modern wound dressing, such as the ability to absorb wound exudate, maintain a wound moisture environment, non-adhesive, painlessness when removed, and providing cooling and soothing effect [10,11]. In fact, the porosity of hydrogel offers tremendous benefits in drug delivery applications especially in sustained release systems, which are controlled via diffusion, swelling or chemical environment [12]. Hydrogels have been applied to many fields as pharmaceutical products, biomedical implants, tissue engineering and regenerative products [9]. Generally, hydrogel can prepare via physical or chemical crosslink. Firstly, physical crosslink technique obtained physical gel that can be reversed because the involved forces were hydrophilic interaction, electrostatic and hydrogen bonding between polymer chains, nevertheless this technique were cheap and easy to prepare. In addition, most crosslinking agents used in this technique are nontoxic. Secondly, chemical crosslink technique acquired irreversible or permanent hydrogel since this method used covalent bonding between polymer chains via the additional small crosslinker molecule, polymer-polymer conjugation, enzyme catalyzed reaction and photosensitive agents. Moreover, most chemical crosslink agents are toxic and expensive [13]. Nevertheless, the most challenging part in preparing hydrogel containing BC is BC dispersion due to the hydrogen bonding between cellulose fibers. Thus, the addition of polyethylene glycol (PEG) solution in BC could enhance BC dispersion in an aqueous phase, since PEG could form a hydrogen bond with BC fiber [14]. Truly, BC alone has no antimicrobial effect. Conversely, BC can prevent bacterial penetration to the wound and can be improved by adding the active drug to the biopolymer to make desired effect [5]. Many antiseptic agents are widely used for preventing bacterial infection, for instance chlorhexidine, triclosan, povidone-iodine and polyhexamethylene biguanide (PHMB) [15].

PHMB, a highly effective antimicrobial agent, is a strong base and positive charge compound that can bind to the phosphate head group on the bacterial cell membrane, leading to potassium ions and other components leakage and resulting in cell death [16,17,18]. PHMB shows good effects against bacteria that cause skin infection, such as *Pseudomonas aeruginosa, Streptococcus pyrogenes* and *Staphylococcus aureus*. PHMB usage is not only in the medical field but also in cosmetics and the environment as a water disinfectant [18] due to its chemical stability, low cytotoxicity, and inexpensiveness. This agent could significantly promote wound healing, unlike other well-known antiseptic agents [16] Accordingly, these polymers are segregated in endosomes, preventing the mammalian nucleus from its harmful effect, which will affect cell division [19].

Hence, this study focused on seeking an optimal hydrogel formulation via physical crosslink technique with a good antibacterial activity using BC, alginate, and pectin for fabricating hydrogel with CaCl_2_ solution, which is nontoxic physical crosslinking agent, then added PHMB, which had good antibacterial activity and a positive charge that can form ionic interaction with COO^−^ of alginate or pectin, similar to Ca^2+^. We hypothesized that the combination of these polymers could develop the hydrogel’s properties with an antimicrobial effect for wound dressing application.

## 2. Materials and Methods

### 2.1. Materials

Bacterial cellulose (BC, white freeze-dried powder with fiber dimensions 50–100 nm) was purchased from CelluloseLab, New Brunswick, Canada. Calcium chloride (CaCl_2_) was purchased from RCI Labscan Ltd., Bangkok, Thailand. Low methoxy pectin (P; degree of esterification = 29%) was purchased from Cargill^TM^, Saint Germain, France. Polyethylene glycol 1500 (PEG; molecular weight 1500 g/mol) was purchased from Tinnakorn Chemical and Supply Co., Ltd., Bangkok, Thailand. Polyhexamethylene biguanide (PHMB) was purchased from Carbosynth Ltd., Compton, UK. Sodium alginate (A) was purchased from Qingdao Bright Moon Seaweed Group Co., Ltd., Qingdao, China. Deionized water (DI) served as a solvent for preparing hydrogels.

### 2.2. Preparation of Hydrogels Containing BC

Hydrogels containing BC were formed by separately preparing 2% *w*/*v* of BC, 0.5% *w*/*v* of A, 5% *w*/*v* of P, and 0.5% *w*/*v* of A + 0.5% *w*/*v* of P (A/P). BC freeze-dried powder was dispersed in 40% *w*/*w* polyethylene glycol using the homogenizer (IKA T25 Ultra-Trurrax, IKA laboratory technology, Staufen, Germany) at 6000 rpm for 10 min. A and P were dissolved in DI water at the concentration of 0.5% *w*/*v* and 5% *w*/*v*, respectively. Then, BC was mixed with A or P or A/P as a weight ratio at 30:70. After that, the blend weighted 10 g was fabricated to form hydrogels by casting on a Petri dish plate and using 3% *w*/*v* of calcium chloride (CaCl_2_) solution as a crosslinking agent. After 2 h, crosslinked hydrogels were taken out and washed with DI water to remove excess Ca^2+^. Then, a filter paper was used to absorb excess water on the hydrogels’ surface, followed by a freeze-drying process (Christ Beta 2–8 LD-plus, Osterode am Harz, Germany) for 24 h, then BC composite sponges were obtained. After that, another three formulations were prepared following the steps expounded above and incorporated 20% *w*/*v* PHMB using the immersion technique for 24 h before the freeze-drying process. The compositions of hydrogel formulations are presented in Table 1.

### 2.3. Hydrogel Containing BC Characterizations

#### 2.3.1. Morphological Characterizations

A morphological study of hydrogels containing BC was carried out using scanning electron microscopy (SEM) (JEOL JCM-7000 NeoScope™ Benchtop, Tokyo, Japan) at 15 kV under low vacuum mode. Hydrogels containing BC formulations were fixed on aluminum stubs with double-sided adhesive carbon tape, then coated with gold for 1 min before taking a cross-sectional image at 50× magnification level.

#### 2.3.2. Thickness and diameter

The average thickness and diameter of hydrogel containing BC were measured using an outside micrometer (3203-25A, Insize Co, Ltd., Suzhou, China). Random samples of individual hydrogel formulations were measured to calculate the average thickness and diameter with their standard deviation (S.D.). Each sample was measured in triplicate.

#### 2.3.3. Mechanical Strength Test

The mechanical strength of hydrogel containing BC was determined using a texture analyzer, TX. TA plus (Stable Micro Systems, Surrey, UK). The hydrogel sample was placed on a plate with a 9.0 mm diameter cylindrical hole. A 2 mm diameter cylindrical stainless probe with a plane flat surface was used at a speed of 2.0 mm/s. The probe passed through the hydrogel at a constant speed until the hydrogel was broken, and peak force (N) was recorded. Each formulation of hydrogels containing BC had at least three repeats. Mechanical strength was reported by puncture strength.

#### 2.3.4. Fluid Uptake Ability

The fluid uptake ability of bacterial cellulose composite sponges was examined by cutting each sample into 1.5 × 1.5 cm^2^ pieces and weighting their dry weight (*Wd*). An individual sample was immersed in a beaker containing 20 mL phosphate buffer saline (PBS) pH 7.4 and incubated at 37 °C. At optimized intervals, wet hydrogels were taken out from PBS, then excess surface PBS was removed by gently blotting with a filter paper. After that, the wet weight of wet hydrogel (*Ws*) was measured. Weights of wet hydrogels were recorded until reaching swelling equilibrium. The experiment was carried out in triplicate under the same conditions, and the equilibrium of fluid content was calculated.

$$\mathrm{Equilibrium}\mathrm{of}\mathrm{fluid}\mathrm{content}\;(\%)=\frac{\left(Ws-Wd\right)}{Ws}\times 100$$

#### 2.3.5. Water Retention Property

The water retention property of bacterial cellulose composite sponges was obtained by immersing dry hydrogel in PBS. After 24 h, wet hydrogels were withdrawn from the PBS and wiped with a filter paper to remove excess surface PBS, then the initial wet weight (*W*0) was recorded. Wet hydrogels were transferred in Petri dishes at room temperature. After that, wet hydrogels (*Wt*) were measured at regular time intervals for 24 h and calculated water retention capacity (%) using the following equation. These tests were carried out in triplicate.

$$\mathrm{Water}\mathrm{retention}\mathrm{capacity}\;(\%)=\frac{Wt}{W0}\times 100$$

#### 2.3.6. Integrity Value

Bacterial cellulose composite sponges were cut into a 1.5 × 1.5 cm^2^ size and their initial weight (*Wi*) was measured. An individual sample was placed in an Erlenmeyer flask containing 30 mL PBS and jolted on a shaker (Eberbach Co., Ann Arbor, MI, USA) at 50 rpm for 24 h. After that, all samples were removed and then, placed into a petri dish, and dried in an oven (Memmert GmbH Co., KG, Schwabach, Germany) at 70 °C. Dried weight (*Wd*) was measured after hydrogel had been completely dried. Experiments were performed in triplicate and the integrity value was defined using the following equation.

$$\mathrm{Integrity}\mathrm{value}\;(\%)=\frac{Wd}{Wi}\times 100$$

#### 2.3.7. Swelling Ratio

The randomly selected three samples of bacterial cellulose composite sponges were cut into 1.5 × 1.5 cm^2^ pieces and their dry weight (*Wd*)was measured before being immersed in 20 mL of PBS pH 7.4 at room temperature for 24 h. After that, all swollen hydrogels were carried out from the PBS, then gently blotted with a filter paper to remove excess PBS, and the wet weight of swollen hydrogels (*Ws*) was measured. The maximum swelling degree (%) was calculated.

$$\mathrm{Maximum}\mathrm{swelling}\mathrm{degree}\;(\%\mathrm{MSD})=\frac{\left(Ws-Wd\right)}{Wd}\times 100$$

### 2.4. Fourier-Transform Infrared Spectroscopy

Chemical interactions of A, BC, P, bacterial cellulose composite sponge formulations and PHMB were performed by Fourier transform infrared (FTIR) spectrometer (FT/IR-4700, Jasco, Tokyo, Japan). All samples were scanned at a resolution of 4 cm^−1^ in transmittance mode from 400–4000 cm^−1^.

### 2.5. PHMB Loading Content

Three random samples of each bacterial cellulose composite sponge incorporated PHMB formulation were added into vials containing 10 mL of deionized water and sonicated using a sonicator (Elmasonic S100H, Elma, Singen, Germany) for 1 h. All solution samples were filtered through a 0.45 μm membrane filter to remove some small hydrogel pieces and then diluted. The average amount of PHMB was analyzed with a UV-spectrophotometer (UV2600i, Shimadzu Corporation, Kyoto, Japan) at 236 nm. The contents of PHMB were calculated from the standard of PHMB solution in a concentration range of 5.0–17.5 μg/mL, with a high linear regression (r^2^ = 0.999). The drug content of the hydrogel incorporated PHMB was determined.

$$\mathrm{PHMB}\mathrm{loading}\mathrm{content}\;(\%)=\frac{The\;amount\;of\;drug\;in\;hydrogel}{Theorectical\;drug\;content}\times 100$$

### 2.6. In Vitro Drug Release Profile

The selected bacterial cellulose composite sponges weighted approximately 1.5 g were created in an in vitro PHMB release profile in a 100 mL beaker containing 50 mL of phosphate buffer saline pH 7.4 and Tris-HCl buffer pH 7.4 at 37 ± 0.5 °C and then stirred continuously with a magnetic bar at 50 rpm. A total of 5 mL of the dissolution media was taken out to measure the amount of released drug (%) at 0.5, 1, 2, 3, 4, 6, 8, 10, and 24 h, and 5 mL of PBS was replaced. A PHMB release was performed using a UV-spectrophotometer (UV2600i, Shimadzu Corporation, Kyoto, Japan) at 236 nm. The selected hydrogel was examined in triplicate, and the amount of released PHMB was calculated using the equation below.

$$\mathrm{Amount}\mathrm{of}\mathrm{released}\mathrm{drug}\;(\%)=\frac{The\;amount\;of\;released\;drug\;at\;specific\;time}{Amount\;of\;drug\;in\;hydrogel}\times 100$$

The drug release profile was expressed via different kinds of drug release kinetic models. Additionally, the first 60% of drug release data was fixed with zero-order, first-order, Higuchi, and Korsmeyer–Peppas models in order to evaluate the most appropriate model of PHMB release from hydrogel.

Firstly, the zero-order model explains the constant dissolution rate over the period of time. Moreover, this model is not related to the drug concentration, however, the rate-limiting step in the zero-order kinetic is time. Zero-order model release can be calculated using the following equation.

Q_t_ = Q_0_ + *k*_0_*t*

where Q_t_ is the released drug accumulation at each predetermined time; Q_0_ is the initial amount of the drug; *k*_0_ is the zero-order kinetic constant; and *t* is time.

Secondly, the first-order kinetic is the drug release profile that depends on drug concentration, so the rate limiting step in the first-order kinetic is the initial drug concentration. First-order kinetic release can be calculated using the following equation.

logQ_0_ − logQ_t_ = *k*_1_*t*/2.303

where Q_t_ is the released drug accumulation at each predetermined time; Q_0_ is the initial amount of the drug; *k*_1_ is the first-order kinetic constant; and *t* is time.

Thirdly, the Higuchi model explains the released drug from the insoluble matrix; on the other hand, the Higuchi model studies the drug release rate of water-soluble or slightly water-soluble drugs incorporated in solid and/or semi-solid matrixes. The Higuchi model can be determined using the following equation.

$$Q_{t}=k_{H}t^{\frac{1}{2}}$$

where Q_t_ is the released drug accumulation at each predetermined time; *k*_H_ is the Higuchi kinetic constant; and *t* is time.

Lastly, the Korsmeyer–Peppas model describes the drug release from a polymer matrix, which is related to the exponential drug release and the fractional drug release. The Korsmeyer–Peppas model can be calculated using the following equation.

Q_t_/Q_0_ = *kt*^n^

where Q_t_/Q_0_ is the fraction of drug release; *k* is the geometrical and structural constant; *t* is time; and n is the release exponent.

### 2.7. Antimicrobial Test

Two selected bacterial species, i.e., *Staphylococcus aureus* ATCC 25923 and *Pseudomonas aeruginosa* ATCC 27853 were grown in Brain Heart Infusion (BHI) medium (HiMedia, Mumbai, India). Isolated bacterial cultures were maintained at 37 °C under aerobic conditions. An overnight culture of *S*. *aureus* and *P. aeruginosa* were determined for the optical density at 600 nm wavelength (OD600) using a spectrophotometer (Beckman Coulter, Fullerton, CA, USA) prior to the preparation of bacterial stock for spreading on BHI agar plates.

Next, hydrogels containing BC incorporated with 0.2% PHMB were determined for antimicrobial activities using the disc diffusion technique. A 0.2% of PHMB was chosen in order to compare it with commercial wound dressing with PHMB [20]. Briefly, BC/A-PHMB was placed on prepared bacterial agar plates. BC/A without PHMB was used as a negative control. Whatman^®^ antibiotic assay discs (GE Healthcare, Pittsburgh, PA, USA) loaded with 20 μL of 20% PHMB solution served as a positive experimental control. Bacterial agar plates with tested hydrogel films were incubated at 37 °C under aerobic cultivation overnight until bacterial lawns were clearly visible. After 24 h of the incubation period, the diameter of an inhibition zone was measured using a Mitutoyo^®^ Digimatic caliper (Mitutoyo Corporation, Kanagawa, Japan). Three independent experiments in triplicate were performed.

### 2.8. Cell Culture

A human keratinocyte cell line (HaCaT) was cultured in Dulbecco’s Modified Eagle’s Media (DMEM) supplemented with 1% penicillin-streptomycin and 10% fetal bovine serum (FBS) and then maintained at 37 °C in a humidified incubator containing an atmosphere of 5% CO_2_.

### 2.9. Cell Viability Assay

The cell viability assay of bacterial cellulose composite sponge was performed using 3-(4,5-dimethylthiazol-2-yl)-2,5-diphenylte-trazolium bromide (MTT) (Sigma-Aldrich, Saint Louis, MO, USA). HaCaT cells were seeded in 96-well plates at a density of 8 × 10^3^ cells per well and incubated for 24 h in a culture medium. The bacterial cellulose composite sponge samples (BC/A and BC/A-PHMB) were cut into 2 × 2 × 2 mm^3^ pieces and soaked in DMEM for 48 h. The solutions of hydrogel extract were filtered through a 0.22 μm membrane filter and incubated with cells for 24 h at 37 °C, 5% CO_2_. After incubation, samples were removed. MTT at a concentration of 0.5 mg/mL was added (100 µL/well) and incubated at 37 °C for 2 h. Then, the medium was replaced with DMSO (100 µL) to solubilize the formazan product. The absorbance for each well was measured at 550 nm, using a microplate reader (Spectramax M3, Molecular Devices, San Jose, USA). The percentage of cell viability was calculated by the following equation.

$$\mathrm{Cell}\mathrm{viability}\;(\%)=\frac{A550\;treatment}{A550\;control}\times 100$$

### 2.10. Statistical Analysis

All results were shown as mean ± standard deviations (S.D.). One-way ANOVA test determined the significant difference using SPSS software version 17.0 (IBM Corporation, Armonk, NY, USA) to analyze the statistical significance of the results when the *p*-value is less than 0.05.

## 3. Results and Discussion

### 3.1. Preparation and Morphological Characterization of Hydrogel Containing BC

**Figure 1:**
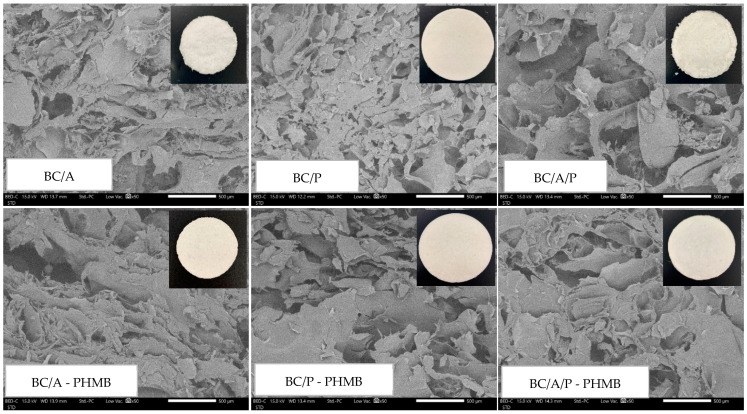
Cross section of sponges composed of BC and incorporated with or without PHMB using SEM at 50×.

Freeze-dried BC was dispersed in 40% *w*/*w* PEG. Moreover, the benefit of adding PEG is to make BC disperse easily and to increase hydrogels’ flexibility [14]. A total of 2% *w*/*v* of BC combined with A or P or A/P was prepared by a casting method adding 3% *w*/*v* CaCl_2_ solution as a crosslinking agent. From our previous study, 0.5% *w*/*v* of A, including 5% *w*/*v* of P alone, cannot form a strong hydrogel [21]. However, these are the lowest concentrations that can form a strong, beautifully shaped hydrogel, when mixed with BC 2% *w*/*v*. This indicates that BC is a good supportive structure [22]. BC hydrogels have also been fabricated by soaking BC sheets in 1% *w*/*v* alginate and using 6 metal cations to form the hydrogel [22]. Li et al. combined alginate and CaCl_2_ solution in Hestrinand Schramm (HS) medium with *K. xylinus* that produced BC into an HS medium. After 10 days, BC hydrogels were obtained [23]. This method was similar to that of Lopez et al., however, Lopez et al. used pectin instead of alginate and different cultivation times [24]. In this study, BC/A and BC/A/P spontaneously shrank when crosslinked with a CaCl_2_ solution due to the high degree of crosslinking of COO^−^ from alginate with Ca^2+^ [25]. After that, PHMB was added as an antimicrobial agent to the formulation. Obviously, the benefit of preparing hydrogel by our method is to reduce the preparation time, which is only one day and a half. Visual examination of all hydrogel formulations shows some differences. The color of BC/A and BC/A/P is white, while BC/P is slightly yellow due to the natural pectin color, resulting in the formulation with PHMB. BC/A and BC/A/P showed a fluff surface; however, after adding PHMB, the surface was smooth since PHMB might form an ionic interaction and a hydrogen bond, which helped hydrogel components to more strongly attach [26]. The diameter and thickness of BC/A-PHMB and BC/A/P-PHMB decreased significantly after soaking in PHMB for 24 h compared to its formulation (BC/A and BC/A/P) due to structural shrinkage while crosslinked with PHMB, shown as diameter and thickness in Table 2. After freeze-drying, all hydrogels look similar to a sponge, and after using SEM, the structure of bacterial cellulose composite sponge formulations revealed numerous porous structures with no significant difference (Figure 1), so numerous porosities inside the hydrogel structure can absorb a great amount of water.

### 3.2. Mechanical Properties of Hydrogels Containing BC

To investigate the influence of PHMB on the mechanical properties of hydrogels containing BC, puncture strength was evaluated, as shown in Table 2. An ideal wound dressing hydrogel must have a high puncture strength to provide its structure when used and to maintain hydrogel durability through application time. Moreover, puncture strength indicates robustness of hydrogel, which influences some properties of hydrogel, such as integrity value and maximum swelling degree. The formulations composed of alginate had a higher puncture strength than those without alginate as their structure shrank when crosslinked with CaCl_2_ solution, which made the composition in the formulation denser [25]. The puncture strength value of all formulations with PHMB increased when compared to all hydrogel formulations without PHMB. BC/A/P-PHMB shows the highest puncture strength (4.50 N/mm^2^), while BC/P shows the lowest (1.75 N/mm^2^). The puncture strength of all formulations incorporating increased PHMB might be the interaction between A or P with PHMB, which might form a charge interaction between the amine group of PHMB and the carboxylic group of A or P, which make the structure of the hydrogel stronger. Our study suggested that PHMB used as a crosslinking agent with CaCl_2_ can increase the material’s strength.

### 3.3. Fluid Uptake Ability, Water Retention Property, Maximum Swelling Degree, and Integrity Value of Hydrogels Containing BC

**Figure 2:**
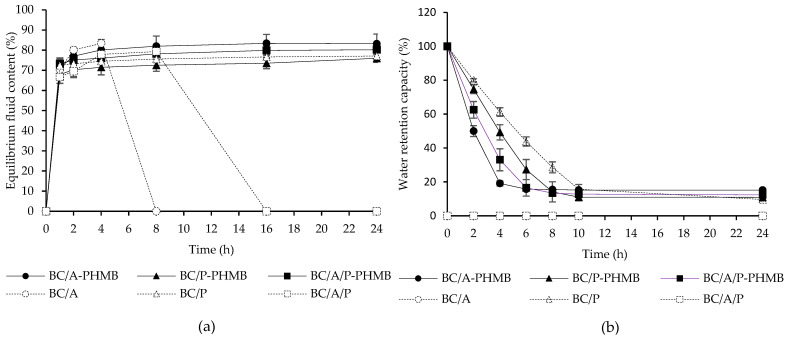
Equilibrium fluid content (**a**), water retention property (**b**) of hydrogels containing BC.

The ideal properties of a wound dressing are absorbing excess exudate, allowing gas exchange, promoting the wound healing process, and keeping their shape when exposed to wound exudates. Hence, evaluating fluid uptake ability, water retention properties, maximum swelling degree (MSD), and integrity value are important tests to find a good candidate for wound dressing materials. Additionally, fluid uptake ability, water retention properties, and MSD are dependent on the porosity and hydrophilicity of the hydrogel since water and biological fluids can pass though the structure of the hydrogel, form a hydrogen bond with the material, and remain in the hydrogel structure [27]. A hydrogel’s fluid uptake ability is an important parameter to evaluate the absorption of excess wound exudates and fluids. In this study, the fluid uptake ability of hydrogel containing BC with or without PHMB was evaluated by incubating in PBS at 37 °C. Figure 2a shows the kinetics of fluid uptake of the hydrogel. Fluid uptake ability tended to increase until the equilibrium state of fluid uptake was achieved after 8 h, however, BC/A and BC/A/P are completely segregated after 4 and 8 h, consequently, because of the degradation of alginate in a buffer solution [28]. Interestingly, BC/A-PHMB and BC/A/P-PHMB are not segregated when PHMB was added into BC/A and BC/A/P. Equilibrium fluid content at 24 h of BC/P, BC/A-PHMB, BC/P-PHMB, and BC/A/P-PHMB was 77.11 ± 1.38, 83.34 ± 4.81, 75.95 ± 1.98, and 80.23 ± 2.48%, respectively. There was a significant difference in equilibrium fluid content at 24 h between BC/A-PHMB with BC/P and BC/P-PHMB at 24 h. BC/A-PHMB showed the highest fluid uptake ability because alginate is a good gelling agent and a high water absorption material due to several carboxyl and hydroxyl groups in the molecule [28], when compared with pectin.

The water loss from hydrogel containing BC when exposed to air was reported as water retention capacity, shown in Figure 2b. Hydrogels lose their water content when exposed to air over long periods under dry conditions. Our study showed that hydrogel composed of BC and P (BC/P, BC/P-PHMB) significantly exhibited greater water retention capacity, when compared to the hydrogel containing BC and A (BC/A-PHMB, BC/A/P-PHMB) in the first 4 h. While BC/A and BC/A/P are completely segregated when immersed in PBS for 24 h, the water retention capacity of these two formulations cannot be evaluated; however, after incorporation with PHMB (BC/A-PHMB and BC/A/P-PHMB), it can be calculated. This result explained that adding PHMB could make the hydrogel structure more rigid and stable. Water retention capacity has shown no significant difference among BC/A-PHMB, BC/P-PHMB, BC/A/P-PHMB, and BC/P over a period of 24 h.

The swelling properties of hydrogel containing BC were evaluated in terms of maximum swelling degree (MSD) after being soaked in PBS for 24 h. Nevertheless, Li et al. formulated BC/alginate hydrogel, which exhibited 613% MSD after 16 h, since their hydrogel was prepared by forcing 0.75% *w*/*v* alginate into BC porous sheet; moreover, the less concentration of alginate that was used, the less%MSD that was achieved [23]. The formulations containing alginate showed higher%MSD than the formulations with pectin because the alginate backbone contains numerous hydroxyl and carboxyl groups which can absorb a large amount of water [28]. Our study showed that BC/A-PHMB exhibited the highest%MSD (407%), while BC/P showed the lowest (309%) (Table 3).

Hydrogel must be able to hold its shape after contact with wound exudates or bodily fluids because one of the problems of swelling hydrogels is the lack of structural integrity; on the other hand, low integrity value hydrogel usually degrades during application. Generally, integrity value indicates durability of hydrogel during implementation [29]. Thus, the integrity value of hydrogels containing BC was measured after being soaked in PBS for 24 h. Our results found that the integrity value of all formulations increased when PHMB added, as shown in Table 3. The integrity value of BC/P, BC/A-PHMB, BC/P-PHMB, and BC/A/P-PHMB was 31 ± 0.50, 55 ± 0.80, 49 ± 0.82, and 50 ± 1.06%, respectively. Thus, PHMB can increased the structural integrity of hydrogels. Moreover, BC can also increase structural integrity since BC is a good supportive structure.

After SEM examination, each hydrogel formulation revealed numerous porosities with no significant difference, so fluid uptake ability and MSD in this study depends on the hydrophilicity of the polymer, which is alginate and pectin including BC; however, the amount of BC in each formulation is the same, hence fluid uptake ability and MSD are influenced by alginate and pectin. Additionally, the water holding capacity of hydrogel is related to the hydroxyl and the carboxylic groups of polymers, as these two functional groups are able to form a hydrogen bond with water.

However, BC/A and BC/A/P are unable to evaluate maximum swelling degree and integrity value, as their structure completely segregated after 24 h of soaking because of the degradation of alginate in phosphate buffer saline, which oxidized the hydroxyl group into the aldehyde group, leading to the bonding between carbon molecules in uronate residue cleavage, resulting in breaking the hydrogel structure [28]. Moreover, this degradation is unpredictable and related to the high molecular weight of alginate. Another reason is phosphate ion in PBS, which is able to competitively bind with calcium ions in crosslinking hydrogel, resulting in the dissolution of calcium ion into the medium [30]. However, after added PHMB, the structural integrity and MSD increased because the amine group of PHMB can interact with the ionic linkage with the carboxylic group, as well as the hydrogen bond with hydroxyl and carboxyl groups of alginate or pectin; on the other hand, PHMB can increase hydrogel strength, which prevented hydrogel segregation, resulting in improved structural integrity and water holding capacity. Additionally, the ion interaction among the amine group and Ca^2+^ with COO^−^ of alginate or pectin can be called a dual crosslinking.

### 3.4. Fourier Transform Infrared Spectroscopy

**Figure 3:**
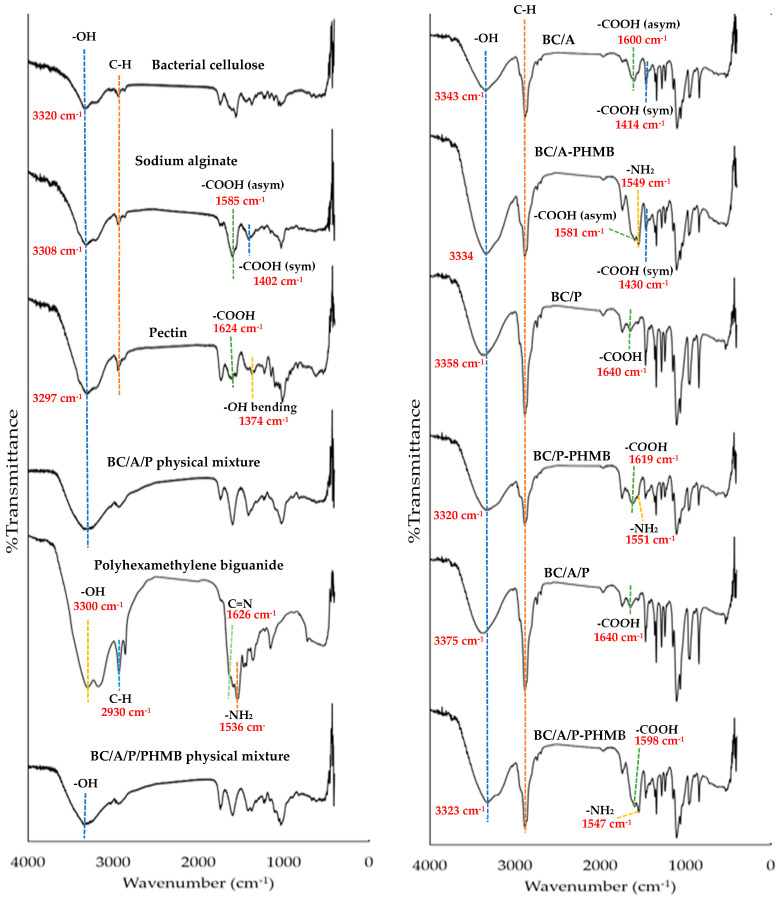
Fourier-transform infrared spectroscopy (FTIR) spectra of A, P, BC, a physical mixture of A, P, and BC with or without PHMB and bacterial cellulose composite sponge formulations.

FTIR analysis was examined to indicate the possible interactions of hydrogels by investigated intensity change or chemical shift, which related to the change of bond order; moreover, the manifest interaction of -OH groups corresponded to hydrogen bonds found based on OH peak change in region 3170–3560 cm^−1^ [31]. The FTIR spectra of starting materials (BC, A, P, PHMB powder), their physical mixture, and all bacterial cellulose composite sponge formulations are shown in Figure 3. In the spectra of sodium alginate (A), the wide band at approximately 3308 cm^−1^ shows the stretching vibration band of the hydroxy (-OH) group. The band at approximately 2936–2855 cm^−1^ relates to the vibration of the C-H bond. The peak band at 1585 and 1402 cm^−1^ relates to the asymmetric and symmetric stretching band of the carboxylic (-COOH) group [32,33]. The spectra of pectin (P) and the observed bands at 3297, 1731, 1624, and 1374 cm^−1^ correspond to -OH stretching, C=O, -COOH asymmetric stretching, and -OH bending, respectively [34,35]. The spectra of bacterial cellulose at 3400 and 2897 cm^−1^ corresponds to -OH stretching and C-H bond stretching, respectively [36]. Generally, the four signature peak bands that are related to the structure of PHMB at 3300, 2930, 1626, and 1536 cm^−1^ corresponded to the stretching vibration of N-H, aliphatic C-H, C=N stretching, and amine (-NH_2_) bending vibration, respectively [37]. In BC/A spectra, sodium alginate’s asymmetric and symmetric stretching of carboxylic (-COOH) groups at 1585 and 1402 cm^−1^ shifted to 1600 and 1414 cm^−1^, respectively, which is related to calcium alginate’s asymmetric and symmetric stretching of carboxylic groups, so it might indicate the interaction between Ca^2+^ and COO^−^ of alginate, which might form an ionic bond during the crosslinking process [32]. Additionally in BC/P and BC/A/P, pectin’s asymmetric stretching of -COOH at 1624 cm^−1^ shifted to 1640 cm^−1^, which might correspond to ionic interaction between pectin’s COOH and Ca^2+^, however alginate’s symmetric stretching of -COOH cannot be identified due to overlapped peaks between alginate’s carboxylic group (1402) and pectin’s bending of the hydroxy group (1374). The amine (-NH_2_) peak of PHMB in BC/A-PHMB, BC/P-PHMB, and BC/A/P-PHMB spectra at 1536 cm^−1^ shifted to 1549, 1551, and 1547 cm^−1^, respectively, moreover -COOH peaks of BC/A-PHMB, BC/P-PHMB, and BC/A/P-PHMB at 1600, 1640, and 1640 cm^−1^ shifted to 1581, 1619, and 1598 cm^−1^, respectively. Additionally, the intensity of the amine (-NH_2_) peak of BC/A-PHMB, BC/P-PHMB, and BC/A/P-PHMB decreased when compared to the amine (-NH_2_) peak of PHMB, thus it might indicate the interaction between PHMB’s amine (-NH_2_) group and the carboxylic (-COOH) group of alginate or pectin. However, the peaks of all starting materials overlapped. Furthermore, the -OH stretching peak of BC/A and BC/P spectra at 3308 and 3297 shifted to 3343 and 3358 cm^−1^, respectively. The BC/A/P -OH peak also shifted to 3375 cm^−1^. In addition, the -OH peak of BC/A-PHMB, BC/P-PHMB, and BC/A/P-PHMB at 3343, 3358, and 3375 shifted to 3334, 3320, and 3323 cm^−1^, respectively, hence the shift of the -OH peak in FTIR spectra might indicate hydrogen bond formation in hydrogel [31].

### 3.5. Drug Content

The PHMB contents (20 mg/hydrogel) in BC/A-PHMB, BC/P-PHMB, and BC/A/P-PHMB were calculated and shown in Table 4. All formulations demonstrated more than 80% drug content, and our result showed no significant difference among the formulations. Theoretically, the amine of PHMB could form an ionic interaction and a hydrogen bond with COO^−^ and a hydroxyl group or a carboxyl group of alginate or pectin, respectively. The drug content in this study is not different from the study of Wiegand et al. (96.2 ± 3.8%) [5].

After examination of the physicochemical properties of hydrogels containing BC with PHMB, there is no significant difference among hydrogel formulations in fluid uptake ability, water retention property, and drug content; however, BC/A-PHMB performed the highest integrity value and maximum swelling degree thus BC/A-PHMB was selected to perform drug release profile, antimicrobial test, and cell viability assay.

### 3.6. PHMB Release Profile

**Figure 4:**
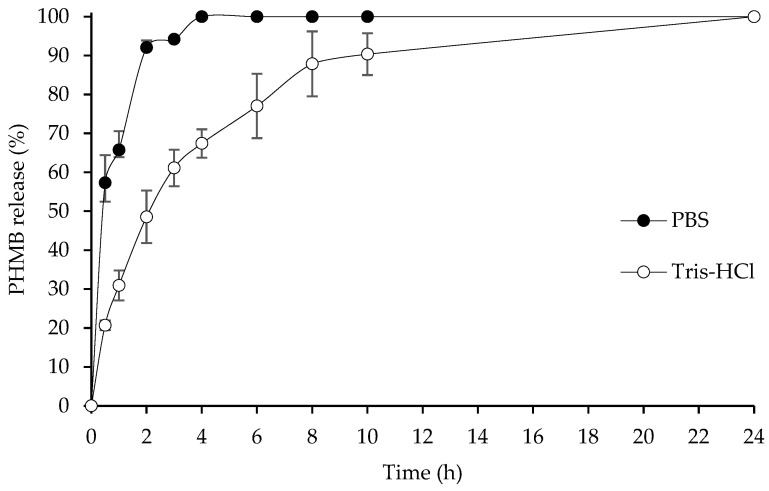
PHMB release profile from BC/A-PHMB hydrogel in PBS and Tris-HCl buffer pH 7.4.

The drug release profile of BC/A-PHMB was determined in both phosphate buffer saline (PBS) pH 7.4 and Tris-HCl buffer pH 7.4, shown in Figure 4. The drug release profile in PBS pH 7.4 showed a significant difference to Tris-HCl buffer pH 7.4 in the first 10 h; however, after 24 h, the drug release profile in both PBS and Tris-HCl reached 100%. In PBS, BC/A-PHMB showed rapid release of PHMB since numerous phosphate ions in PBS can compete with carboxyl groups of alginates to bind amine group of PHMB [16] and Ca^2+^ [38], causing a loose hydrogel structure; in consequence, PHMB unleashes from hydrogel, resulting in PHMB release in PBS achieving 100% after 4 h. On the other hand, the PHMB release profile in Tris-HCl demonstrated a slow-release profile because there is no catalyst similar to phosphate ion to increase the rate of drug release. The rate of controlled drug release depends on drug solubility in the medium, interaction between drug and polymer, and drug penetration through polymer [38]. The drug release profile of PHMB-loaded chitosan/PEO nanofiber in deionized water, prepared by Dilamian et al. tended to increase and achieve approximately 100% at 24 h, which is similar to our result [39].

The drug release profile of PHMB in this study was investigated by various models, including zero-order, first-order, Higuchi, and Korsmeyer–Peppas models, and correlation coefficient (R^2^) and the release rate constant values are shown in Table 5. After fixing with a different type of model, our study found that the most appropriate model to evaluate PHMB released from hydrogel was the Higuchi model because of higher R^2^, so the Higuchi model revealed that the PHMB release mechanism from hydrogel was diffusion from a polymeric matrix [40]. Furthermore, PHMB released in PBS showed a higher Higuchi kinetic constant than in Tris-HCl buffer, which corresponded that PHMB released in PBS is higher than Tris-HCl, resulting in a faster achievement of 100% drug release. Additionally, this experiment was also fixed with a Korsmeyer–Peppas model to clarify the possible release mechanism using a release exponent (n) value. Theoretically, n ≤ 0.45 corresponds to a Fickian diffusion mechanism, 0.45 < n < 0.89 correlates to a non-Fickian diffusion, n = 0.89 corresponds to zero order release (Case II transport), and n > 0.89 correlates to super case II transport [40,41]. In this study, the *n* value of BC/A-PHMB in PBS and Tris-HCl were 0.5906 and 0.5613, respectively, which correlated to non-Fickian diffusion, where the polymer relaxation time was approximately equal to the characteristic solvent diffusion time [42], meaning that active compound release and solvent absorption depend on not only swelling properties of polymer but also polymer/solvent couple viscoelastic properties [14,43].

### 3.7. Antimicrobial Activity

In the disc diffusion test, we loaded 20 μL of 20% PHMB per Whatman disc as a positive control; on the other hand, our study used BC/A as a negative control. The BC/A-PHMB hydrogel, which contained 0.2% of PHMB exerted an inhibition against *S. aureus* and *P. aeruginosa* growth, and there was a slightly significant difference between BC/A-PHMB and a positive control, as shown in Table 6. Our results found that the antibacterial effect of PHMB against *S. aureus* slightly reduced after incorporation within the hydrogel, however, it increased the effect of PHMB against *P. aeruginosa*, which is a gram-negative bacteria. Our results agree with previous findings that topical use of PHMB minimized the bacterial contamination by which the wound healing process is promoted [15].

### 3.8. Cell Viability

**Figure 5:**
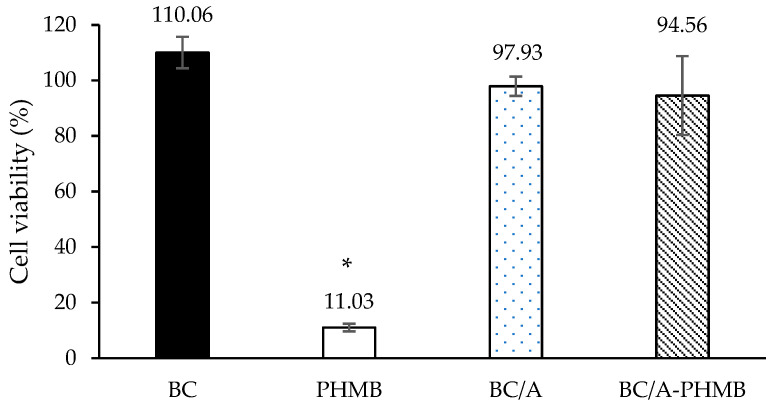
HaCaT cell viability after exposure to BC, PHMB, BC/A, BC/A-PHMB. The results are expressed as mean ± S.D. at a significant level of * *p* < 0.05 in comparison with BC/A.

The effects of hydrogel formulations on human keratinocyte cell viability were determined to ensure that hydrogels were safe for use as a transdermal drug delivery system. The results of the MTT assay can be used to identify the optimal hydrogel formulation that can influence the cell viability of keratinocyte cells. The viability of HaCaT cells treated with BC, PHMB, BC/A, and BC/A-PHMB hydrogel formulations was expressed as a percentage compared to the control, as shown in Figure 5. Cells exposed to BC, BC/A, and BC/A-PHMB had a% cell viability of 110.06%, 97.93%, and 94.56%, respectively. These results suggested that hydrogel formulations composed of BC alone or BC plus alginate were biocompatible with the keratinocyte cell line. In contrast, the viability of HaCaT cells treated with PHMB was reduced to 11.03%, indicating cytotoxicity to human keratinocytes. The addition of bacterial cellulose and alginate significantly reduced the cytotoxicity of PHMB.

## 4. Conclusions

Hydrogels containing bacterial cellulose with alginate and/or pectin loaded PHMB as a drug of water-soluble antibiotic were prepared using CaCl_2_ as a physical crosslinking agent via ionic interaction. Our study found that the hydrogel structure of alginate formulation shrank, while crosslinking with CaCl_2_ made hydrogel composition denser, resulting in enhancing puncture strength and integrity value, however the diameter and thickness of the hydrogel were reduced. The FTIR spectra revealed that the peak of the carboxylic group of alginate and pectin shifted, which indicated the interaction between Ca^2+^ and COO^−^. Additionally, the peak of the amine group of PHMB also shifted, which related to the interaction between NH_3_^+^ and COO^−^ and generated a dual crosslink. Among 6-hydrogel formulations, BC/A-PHMB demonstrated suitable fluid uptake ability, water retention property, maximum swelling degree, and integrity value, with no cytotoxicity to the HaCaT cell, and it exhibited good bacterial activity against *S. aureus* and *P. aeruginosa*. BC/A-PHMB also showed a PHMB content over 80% and demonstrated a prolonged release profile of up to 24 h. Thus, this study concluded that a novel approach for BC/A hydrogel preparation by physical dual crosslinking technique with Ca^2+^ and PHMB could be a potential model for wound dressing application with excellent antibacterial activity. Nevertheless, in a further study, irritation examination should be investigated.

## Figures and Tables

**Table 1:** Composition of different hydrogels containing BC.

Sample Code	Polymer Composition				Crosslinking Agent	Drug
	2% *w*/*v* BC	0.5% *w*/*v* A	5% *w*/*v* P	0.5% *w*/*v* A + 0.5% *w*/*v* P	Ca^2+^	PHMB
BC/A	+	+	−	−	+	−
BC/P	+	−	+	−	+	−
BC/A/P	+	−	−	+	+	−
BC/A-PHMB	+	+	−	−	+	+
BC/P-PHMB	+	−	+	−	+	+
BC/A/P-PHMB	+	−	−	+	+	+

**Table 2:** Thickness, diameter, and puncture strength of hydrogels containing BC.

Formulation	Thickness (mm)	Diameter (mm)	Puncture Strength (N/mm^2^)
BC/A	3.55 ± 0.18 ^a^	31.28 ± 0.15 ^a^	3.18 ± 0.12 ^a^
BC/P	4.65 ± 0.09 ^b^	47.74 ± 0.29 ^b^	1.75 ± 0.10 ^b^
BC/A/P	4.16 ± 0.21 ^c^	35.16 ± 0.74 ^c^	2.51 ± 0.08 ^c^
BC/A-PHMB	3.21 ± 0.10 ^d^	26.32 ± 0.02 ^d^	3.52 ± 0.16 ^d^
BC/P-PHMB	4.38 ± 0.07 ^c^	47.03 ± 0.78 ^b^	2.15 ± 0.15 ^e^
BC/A/P-PHMB	3.41 ± 0.29 ^a,d^	26.68 ± 0.19 ^d^	4.50 ± 0.22 ^f^

For each test, average values with a different letter show significant difference. In contrast, average values with the same letter are not statistically different (*p* < 0.05).

**Table 3:** Integrity value and maximum swelling degree of hydrogels containing BC.

Formulation	Integrity Value (%)	Maximum Swelling Degree (%)
BC/A	0	ND
BC/P	31 ± 0.50 ^a^	309 ± 6.78 ^a^
BC/A/P	0	ND
BC/A-PHMB	55 ± 0.80 ^b^	407 ± 25.87 ^b^
BC/P-PHMB	49 ± 0.82 ^c^	348 ± 11.97 ^c^
BC/A/P-PHMB	50 ± 1.06 ^c^	353 ± 25.12 ^c^

For each test, average values with a different letter show significant difference. In contrast, average values with the same letter are not statistically different (*p* < 0.05). ND indicates that the value was not detected.

**Table 4:** Drug content of BC/A-PHMB, BC/P-PHMB and BC/A/P-PHMB hydrogels.

Formulation	Drug Content (%)
BC/A-PHMB	101.97 ± 6.95 ^a^
BC/P-PHMB	98.10 ± 5.24 ^a^
BC/A/P-PHMB	98.08 ± 4.14 ^a^

For each test, the average values with the same letter are not statistically different (*p* > 0.05).

**Table 5:** BC/A-PHMB released kinetic profile in various drug release models.

Kinetic Models	Parameter	Buffer	
		PBS	Tris-HCl
Zero-order	R^2^	0.8947	0.9645
	*K*_0_ (h^−1^)	15.8502	13.5530
First-order	R^2^	0.8885	0.9011
	*K*_1_ (h^−1^)	0.2101	2.7454
Higuchi	R^2^	0.9845	0.9979
	*K*_H_ (h^1/2^)	61.4814	33.8163
Korsmeyer-Peppas	R^2^	0.9648	0.9981
	*K* (h^−n^)	70.8349	31.8966
	*n*	0.5906	0.5613

**Table 6:** Antibacterial activity of BC/A-PHMB against *S. aureus* and *P. aeruginosa* growth.

Sample	Diameter of Inhibition Zone (mm)	
	*S. aureus*	*P. aeruginosa*
BC/A	ND	ND
20% PHMB	16.00 ± 0.22 ^a^	14.49 ± 0.48 ^a^
BC/A-PHMB	14.28 ± 0.31 ^b^	20.55 ± 1.53 ^b^

For each test, average values with different letters indicate a statistically significant difference (*p* < 0.05). ND is not detected.

## Data Availability

Not applicable.
